# Excreta biomarkers in response to different gut barrier dysfunction models and probiotic supplementation in broiler chickens

**DOI:** 10.1371/journal.pone.0237505

**Published:** 2020-08-13

**Authors:** Reza Barekatain, Gordon S. Howarth, Nicky-Lee Willson, David Cadogan, Stuart Wilkinson

**Affiliations:** 1 South Australian Research and Development Institute, Roseworthy, SA, Australia; 2 School of Animal and Veterinary Sciences, University of Adelaide, Roseworthy, SA, Australia; 3 Feedworks Pty Ltd, Romsey, VIC, Australia; USDA-Agricultural Research Service, UNITED STATES

## Abstract

Increased intestinal permeability (IP) and inflammation are both linked with functionality of the intestinal barrier and in particular enterocytes. Currently, almost all assessment methods of the intestinal barrier function are invasive. The present study aimed to quantify selected proteins as novel biomarkers in excreta of broiler chickens to facilitate non-invasive assessment of gut barrier function using enzyme-linked immunosorbent assays (ELISA). It was further hypothesised that probiotics as feed additives may counteract gut barrier dysfunction. A 3 × 2 factorial arrangement of treatments was used with the main factors being gut barrier dysfunction models (control, rye-based diet, and dexamethasone–DEX) with and without probiotic supplementation (a three-strain Bacillus) using 72 male Ross 308 day-old chickens. Each of the 6 experimental treatments was replicated 12 times. On d 21 of age, fluorescein isothiocyanate dextran (FITC-d) uptake into serum was examined to test IP. Fresh excreta samples were collected on d 20. The biomarkers included alpha-1 antitrypsin (A1AT), intestinal fatty acid binding protein (I-FABP), lipocalin-2 (LCN2), fibronectin (FN), intestinal alkaline phosphatase (IAP), ovotransferrin (OVT) and superoxide dismutase [Cu-Zn] (SOD1). Only DEX increased (*P*<0.001) FITC-d passage to the blood on d 21 of age, indicating a greater IP. The excreta concentrations of A1AT, I-FABP and SOD1 were unaltered by the experimental treatments. DEX increased (*P*<0.05) FN concentration in excreta compared with control birds. Conversely, inclusion of rye in the diet reduced (*P*<0.05) FN but increased (*P*<0.001) OVT in excreta. Independently, DEX decreased IAP (*P*<0.05) in excreta compared with control and rye-fed birds. The excreta concentration of LCN2 tended (*P* = 0.086) to increase in birds injected by DEX. There was no demonstrable effect of probiotic addition on any of the studied parameters. Among the tested biomarkers, FN, IAP, and LCN2 revealed promise as biomarkers of intestinal barrier function quantified by ELISA kits.

## Background

Maintaining and improving gut health is fundamentally important as the gut supports optimal digestion and therefore performance and profitability of production. Managing gut health through barrier function is regarded as a new frontier for disease prevention across different species [1]. In poultry, considerable research has been done on improving animal performance and gut health through various nutritional approaches. However, few objective measures have been identified that could relate to the functionality of the intestinal barrier and detection of inflammation. The complex structure of the epithelium, consisting of a mucus layer covering a single layer of epithelial cells, plays a crucial role in controlling the permeability and selective absorption of nutrients [2]. Disruption of tight junction proteins, alteration in the mucus layer or changes in proliferation of epithelial cells could compromise gut integrity, increase bacterial translocation and eventually cause inflammation. Inflammation often leads to impaired performance that is a significant loss to the poultry industry. In poultry, few biomarkers, related to gut permeability and inflammation, have been identified that could be non-invasive, simple and field-relevant. Biomarker assessment has been limited to invasive methods requiring intestinal tissue sampling or analysis of differentially sized sugars via methods that require birds to be bled and/or euthanized [3]. There have been very recent efforts to identify biomarkers of gut barrier function based on pathogenic and necrotising agents [4]. However, ideal biomarkers should not only be reflective of one particular model and it is important to associate potential biomarkers to other available physiological (i.e. stress) and nutritional models. Besides, even less information is available for excreta biomarkers. Accordingly, intestinal contents, including from the colon, have been used as a proxy to excreta [4].

It is clear that not a single universal biomarker exists for assessing gut health [5], therefore there is a need for a set of objective biomarkers that could be detectable in biological fluids as well as excreta as a primary step to facilitate non-invasive, farm-relevant evaluation of intestinal inflammation and barrier function. Despite the lack of data for poultry, research in humans [6] has identified some faecal biomarkers related to gut inflammation and permeability such as alpha 1-antitrypsin inhibitor (biomarker of gut permeability). Recent reviews also list potential biomarkers that could be detected in excreta [7]. Intestinal fatty acid binding proteins is cytoplasmic protein exclusive to the intestinal enterocytes and can be regarded as a biomarker [8]. Other potential biomarkers include lipocalin 2 [9], ovotransferrin [10], superoxide dismutase [11], fibronectin [4] and intestinal alkaline phosphatase [12]. In most cases, it is unknown how useful excreta biomarkers can be in poultry and even the reference values are lacking. So far little attempt has been made to link these potential biomarkers with inducing agents of gut inflammation and permeability such as stress, bacterial, hormonal and feed-related factors.

Dietary manipulation and feed additives are among strategies to combat gut barrier dysfunction and enteric disorders. Probiotics, in particular, have been shown to help maintain intestinal barrier function through modulating microbiota composition, maintaining permeability, enhancement of immune responses and physical characteristics of the mucous layer [13]. Gadde et al. [14] found that dietary supplementation of *Bacillus subtilis*-based probiotics positively influenced gut barrier integrity by increasing tight junction gene expression. However, little is known in poultry whether any of the excreta biomarkers can be affected by probiotics. If excreta biomarkers are found respondent to a relevant dietary probiotic, this could facilitate rapid and non-invasive assessment of dietary interventions directly linked with intestinal functions.

The current project was not designed to be a nutrition study in which the growth performance of different group of birds would be compared but rather subjecting the individual birds to gut barrier dysfunction models and obtaining replicated excreta samples in which the biomarkers were studied. The project sought to identify a suite of potential biomarkers detectable in excreta of broiler chickens and aimed to test them via the use of poultry models of intestinal barrier dysfunction (gut leakage models) and through the application of feed-related factors. It also aimed to simultaneously investigate possible counteracting effects of probiotic supplementation on the studied biomarkers. Chicken specific reagents of these biomarkers have only recently been made available. Therefore, the potential of ELISA was investigated since the method is relatively rapid and can directly quantify proteins.

## Materials and methods

All experimental procedures were approved by the Animal Ethics Committees of The Primary Industries and Regions South Australia (07/18) and the University of Adelaide (S-2018-065).

The experiment comprised a 2 × 3 factorial arrangement of treatments. The main factors were gut barrier dysfunction models (control, rye-based diet and dexamethasone–DEX) without and with probiotic supplementation of the diets. The probiotic used in the study was a three-strain Bacillus product (Enviva Pro 202 BA, Dansico, Dupont) with minimum activity of 2.5 x 10^9^ colony forming unit/g. The probiotic was supplemented at 60 ppm to the diets. The probiotic was provided by Feedworks Pty Ltd. (Romsey, VIC) and their recommendation was followed for the inclusion rate. Off-sex male Ross 308 day-old chickens (n = 72) were obtained from Aviagen hatchery (Goulburn, NSW) and were brought to the poultry research facility at the Roseworthy Campus of the University of Adelaide. Upon arrival, birds were kept in two groups in raised pens and were given starter diets with or without probiotic supplementation. On day 13 of age, birds were transferred to 72 individual metabolism cages for experimental procedures. Half of the birds received the diets supplemented with probiotic while the remaining were fed un-supplemented diets. The birds that had received the probiotic in the starter phase were maintained on probiotic diet throughout the experiment. Each of the six experimental treatments were replicated 12 times. Individual bodyweights were recorded at the beginning and end of the 8-day period in metabolism cages. Feed consumption was also individually recorded and feed conversion ratio was subsequently calculated. As the individual birds were used in line with the objective of the study, and hence a low number of birds for any performance comparison, the performance results presented in the study were only an indication of the status of birds from which the excreta samples were collected for detection of the biomarkers. All birds were fed *ad libitum* and had access to water via nipple drinkers throughout the experiment. Birds were maintained on 16 hours of light and 8 hours of darkness except for the first 3 days when they were exposed to 23 hours of light. The room temperature was kept at 34 ºC during the first 3 d followed by a gradual decrease to 23 ºC by the end of study at d 21 of age.

Experimental diets were formulated to be iso-nitrogenous and iso-energetic (Table 1). Main ingredients were analysed for nutrient composition using near infrared reflectance (Evonik Industries). Digestible amino acids for rye were the average values obtained from Zuber et al. [15]. A rye-based diet was used as a model of gut barrier dysfunction according to Latorre et al. [16]. Dexamethasone was used as another model based on previous experiments [17, 18]. Birds were provided with experimental diets from d 13 to 21. Birds in the DEX group were injected intramuscularly in the breast with DEX at 0.5 mg/kg body weight on d 14, 16, 18 and 20 of age. The DEX preparation of solution for each injection followed the procedure previously described by Wideman and Pevzner [18].

**Table 1:** Ingredient and nutrient composition of control and rye-based experimental diets.

	Rye-based diet	Control and DEX
**Ingredients (g/kg)**		
Rye	525.5	0.0
Wheat	0.0	652.2
Soybean meal	346.4	262.8
Canola oil	86.8	42.6
Limestone	12.2	11.5
Di-calcium phosphate	14.2	15.6
Sodium chloride	1.0	1.3
Sodium bicarbonate	5.0	4.0
Vitamin and mineral premix^1^	1.6	1.6
Choline Cl 70%	0.5	0.5
L-lysine HCl 78.4%	1.7	3.4
DL-methionine	3.5	2.7
L-threonine	1.6	1.8
**Nutrients (g/kg unless otherwise noted)**		
Dry matter	904.2	899.4
Metabolizable energy (kcal/kg)	3000	3000
Crude protein	215.0	216.2
Crude fat	101.0	58.2
Crude fiber	38.1	24.2
Dig^2^ Arg	13.7	12.3
Dig Lys	11.5	11.5
Dig Met	6.1	5.5
Dig M+C	8.7	8.7
Dig Trp	2.6	2.7
Dig Leu	12.4	12.8
Dig Ile	8.2	8.2
Dig Thr	7.7	7.7
Dig Val	9.0	9.0
Calcium	8.7	8.7
Available phosphorus	4.3	4.3
Sodium	2.0	2.0
Chloride	2.0	2.0

^1^Vitamin and mineral concentrate supplied per kilogram of diet: retinol, 12,000 IU; cholecalciferol, 5,000 IU; tocopheryl acetate, 75 mg, menadione, 3 mg; thiamine, 3 mg; riboflavin, 8 mg; niacin, 55 mg; pantothenate, 13 mg; pyridoxine, 5 mg; folate, 2 mg; cyanocobalamin, 16 μg; biotin, 200 μg; cereal-based carrier, 149 mg; mineral oil, 2.5 mg; Cu (sulfate), 16 mg; Fe (sulfate), 40 mg; I (iodide), 1.25 mg; Se (selenate), 0.3 mg; Mn (sulfate and oxide), 120 mg; Zn (sulfate and oxide), 100 mg; cereal-based carrier, 128 mg; mineral oil, 3.75 mg.

^2^ Digestible

### FITC-d test

On d 21, each bird was administered an aqueous oral gavage solution of FITC-d (2.2 mg/bird) similar to previous studies [17, 19]. After 150 min, blood collection (max. 2 ml) was carried out from the live bird via the jugular vein. Blood samples were kept at room temperature for at least 3 hours to allow clotting. Subsequently, serum samples were separated after centrifuging blood tubes at 1000 g for 15 min at 4°C. The concentration of FITC-d was determined using a Synergy MX plate reader (Biotek Instruments, Bedfordshire, UK) with excitation and emission wavelengths set at 485 and 530 nm, respectively. Standards and samples were analysed in triplicate. On d 21, all birds were then euthanized by cervical dislocation and weights of bursa, spleen and liver were recorded.

### Excreta collection and processing

On the evening of day 20 of age, following the last DEX injection, all excreta trays were cleaned and fresh excreta samples collected for each of the 72 birds within 6 hours. Excreta samples were then stored at -80° C until analysis. The frozen excreta samples were thawed and subsequently diluted (1:10) with PBS. Samples were then thoroughly mixed and then centrifuged at 1500 g for 20 min at 4° C. Aliquots of the supernatants for each samples were then obtained and kept at -80° C until used for assays.

### ELISA assays

Commercial ELISA kits for chicken alpha 1 antitrypsin (MBS028567), intestinal fatty acid binding protein (MBS741864), Lipocalin 2 (MBS005459), fibronectin (MBS778116), intestinal alkaline phosphatase (MBS734160), ovotransferrin (MBS944289) were sourced from MyBioSource (San Diego, CA). The kit for chicken superoxide dismutase [Cu-Zn] (SOD1) was sourced from Wuhan Fine Biotech Co., Ltd. (Hubei, China). All the assays were carried out according to the manufacturer’s instructions. Each blank and standard solution was replicated three times on each plate and samples were assayed in duplicate. Optical densities for all assays were determined using a microplate reader (Bio-Rad Benchmarch Plus^TM^, CA, USA).

### Statistical analysis

All data were subjected to statistical analysis using two-way ANOVA (SAS Statistical package 9.4). The main effects of gut barrier dysfunction and probiotic, as well as their interaction, were assessed. When significant difference was detected, means were separated and compared using Least Square Differences test. Data were checked for normal distribution. For ELISA assays, occasional outliers were removed from the data if there were ± 3× standard deviations from the mean. Each individually housed bird and its respective sample was considered an experimental unit. The level of significance was considered *P* < 0.05 and tendency was considered for 0.05 ≤ *P* ≤ 0.10.

## Results

### Performance of individually-housed birds

Due to the use of individual birds, and therefore a low number, results for performance parameters are presented as indicative of status of birds shown in Table 2. There was no interaction between the gut leakage models and supplementation of probiotic for feed consumption, body weight gain and FCR. No effect of probiotic was also observed for any of the studied parameters. Both rye-based diet and DEX decreased feed intake (*P*<0.0001) compared with the control group of birds. Body weight gain (*P*<0.0001) and FCR (*P*<0.0001) were also compromised most by DEX followed by the rye-based diet compared with control birds.

**Table 2:** Indicative performance of broilers subjected to two leaky gut models with and without probiotic from d 13 to 21 of age^1^^,^^2^.

		Feed intake (g/bird)	Body weight gain (g/bird)	FCR (g feed per g gain)
Main effects				
*Leaky gut model*				
Control (n = 24)		599^a^	452^a^	1.33^c^
Rye-based diet (n = 24)		526^b^	356^b^	1.48^b^
DEX (n = 24)		510^b^	156^c^	3.34^a^
*Probiotic*				
No (n = 36)		543	322	2.042
Yes (n = 36)		547	321	2.056
	SEM^3^	5.59	4.63	0.030
*Source of variation*				
Model		< .0001	< .0001	< .0001
Probiotic		0.77	0.84	0.81
Model × Probiotic		0.08	0.41	0.99

^1^ Means within a column not sharing a superscript ^(a-c)^ differ significantly at the P level shown for the main effects.

^2^ Values in parenthesis represent the number of replicates/bird

^3^ Pooled standard error of the mean (n = 72)

As shown in Table 3, DEX injection severely reduced the weight of spleen (*P*<0.0001) and bursa (*P*<0.0001) and enlarged the liver (*P*<0.0001) compared with rye fed or control groups of birds. Feeding rye reduced the relative weight of liver and increased bursa weight (*P*<0.0001) only, compared with control birds.

**Table 3:** Relative weight (g/100g body weight) of spleen, bursa and liver of broilers subjected to experimental treatments^1^^,^^2^.

		Spleen	Bursa	Liver
Main effects				
*Leaky gut model*				
Control (n = 24)		0.071^a^	0.249^b^	2.68^b^
Rye-based diet (n = 24)		0.072^a^	0.272^a^	2.37^c^
DEX (n = 24)		0.034^b^	0.064^c^	4.17^a^
*Probiotic*				
No (n = 36)		0.059	0.194	3.14
Yes (n = 36)		0.059	0.190	3.01
	SEM^3^	0.0014	0.0051	0.036
*Source of variation*				
Model		<0.0001	<0.0001	<0.0001
Probiotic		0.976	0.723	0.083
Model × Probiotic		0.693	0.068	0.222

^1^ Means within a column not sharing a superscript ^(a-c)^ differ significantly at the P level shown for the main effects.

^2^ Values in parenthesis represent the number of replicates/bird

^3^ Pooled standard error of the mean (n = 72)

### Intestinal permeability

**Fig 1:**
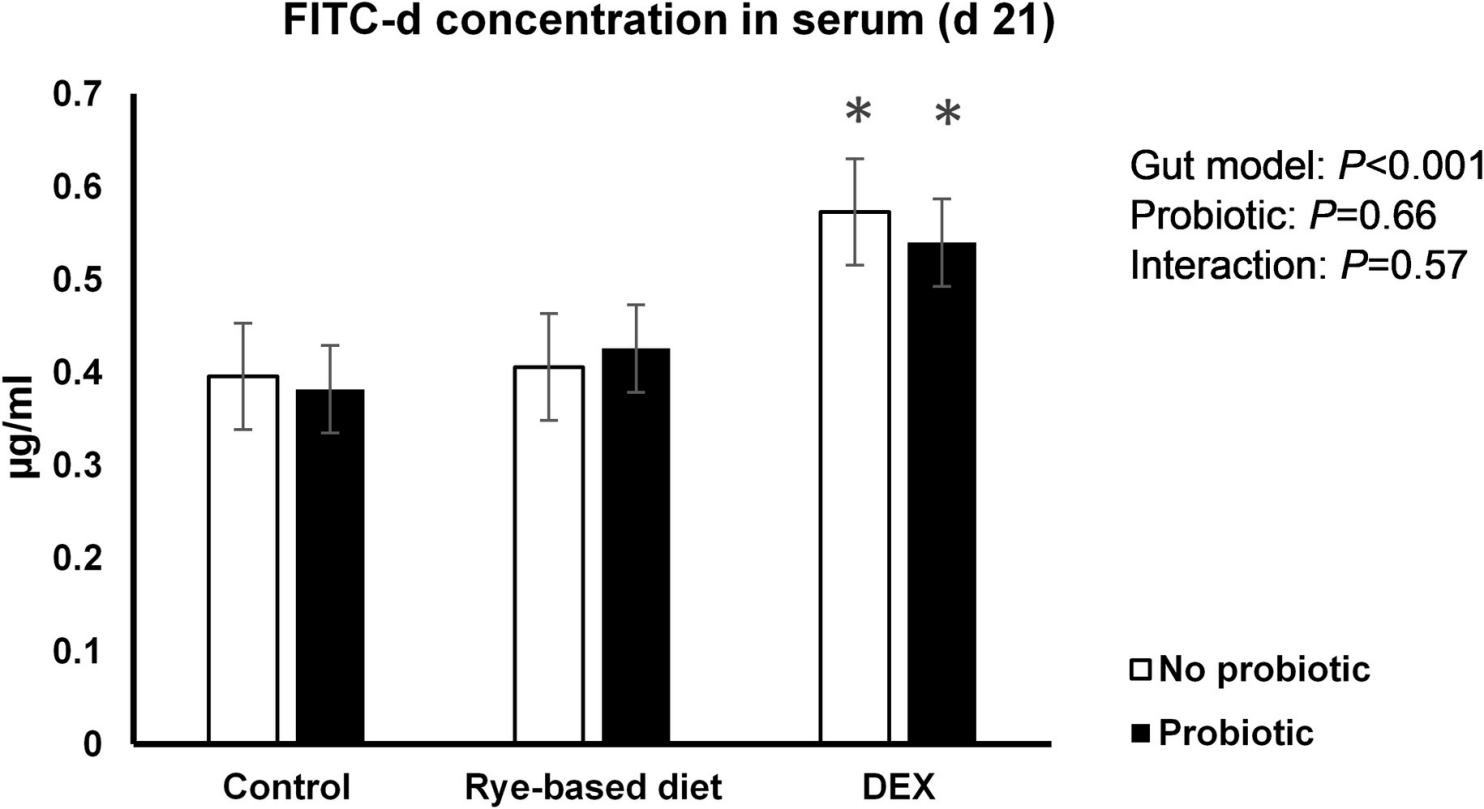
Serum FITC-d concentration of broilers subjected to two leaky gut models with and without probiotic. Error bars represent standard error of the mean (SEM).

The concentration of FITC-d in blood is illustrated in Fig 1. While DEX increased (*P*<0.001) the passage of FITC-d from the intestine into the blood, there was no significant effect of rye inclusion or probiotic.

### Excreta biomarkers

**Fig 2:**
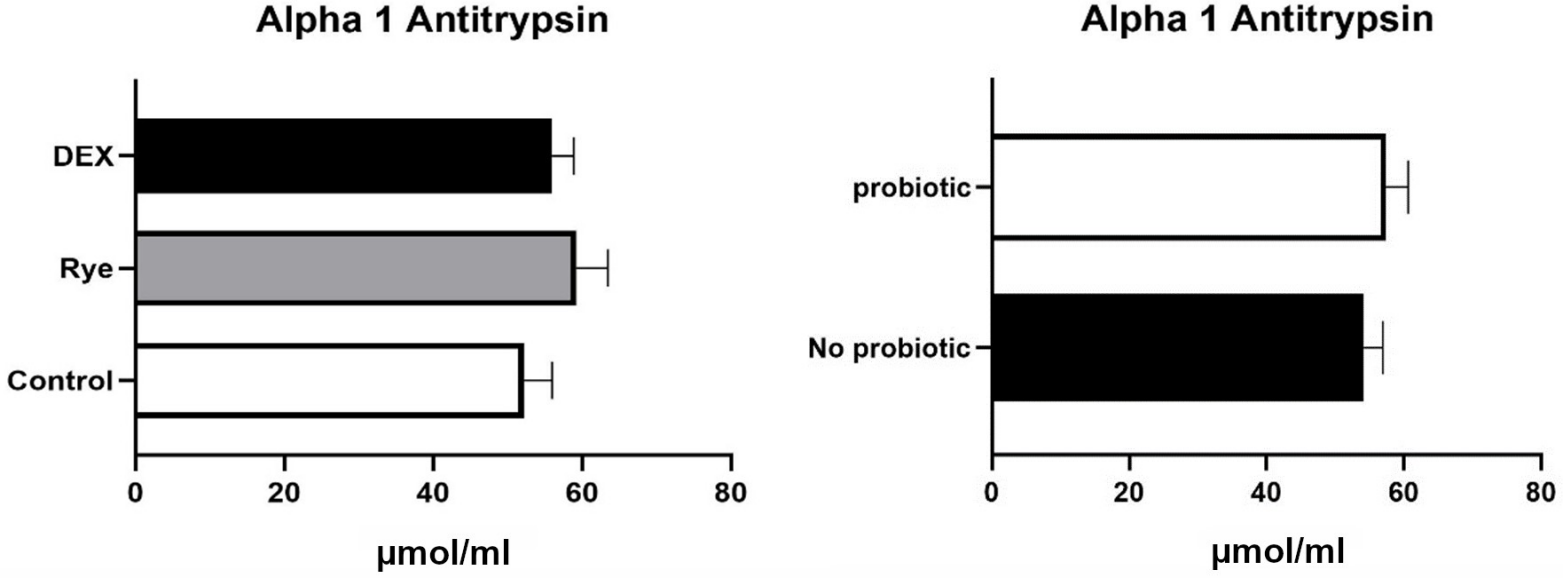
Concentration of Alpha 1 antitrypsin in excreta samples of broilers (n = 72) for the main effects of gut barrier dysfunction models (*P*>0.05) and probiotic supplementation (*P*>0.05). Error bars represent standard error of the mean (SEM).

**Fig 3:**
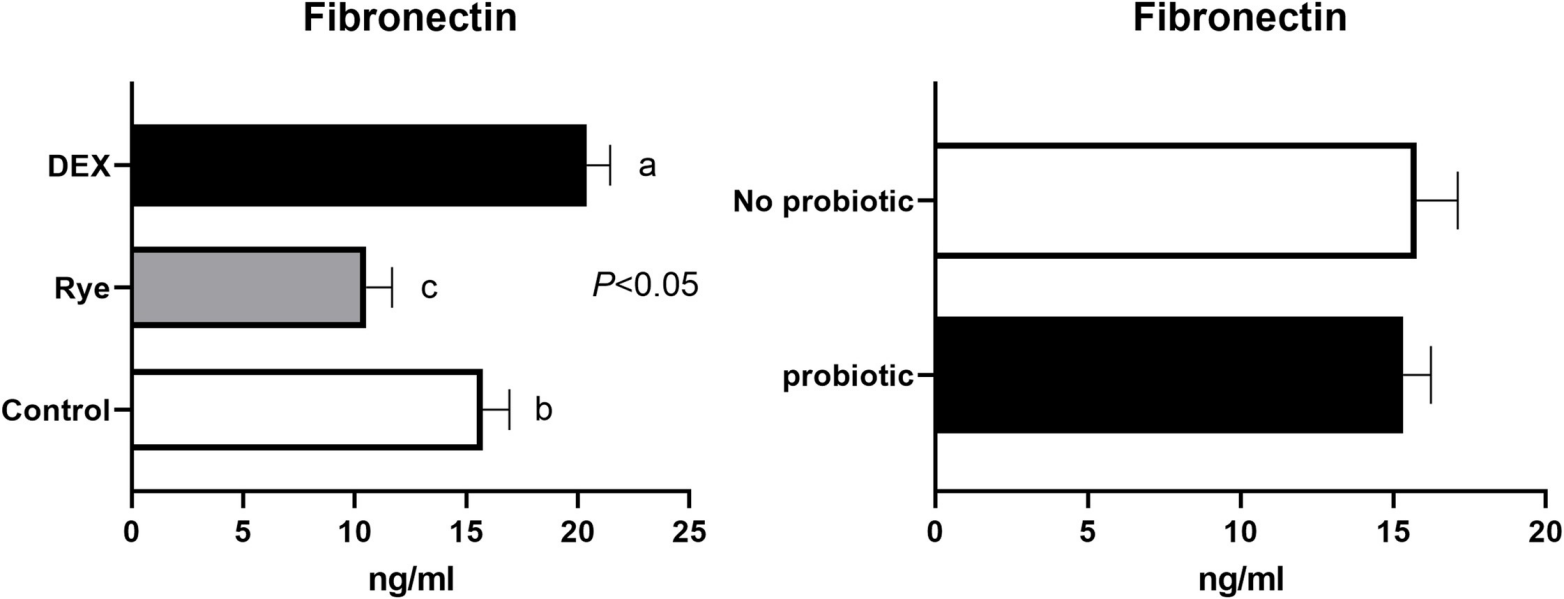
Concentration of fibronectin in excreta samples (n = 72) for the main effects of gut barrier dysfunction models (*P*<0.05) and probiotic supplementation (*P*>0.05). The error bars represent standard error of the mean (SEM). Bars with differing superscripts are statistically different (*P*<0.05).

None of the biomarkers in excreta were affected by dietary supplementation of probiotic and there was no interaction between the challenge models and probiotic. The concentration of alpha 1-antitrypsin assayed by ELISA is shown in Fig 2. With an average concentration of 55.8 μmol/ml in the excreta supernatant, alpha 1-antitrypsin was not affected by any of the experimental factors. As shown in Fig 3, DEX increased (*P*<0.05) fibronectin concentration by 28% (15.7 vs 20.2 ng/ml) compared with control birds. Conversely, inclusion of rye in the diet reduced (*P*<0.05) fibronectin concentration by 25.7%.

**Fig 4:**
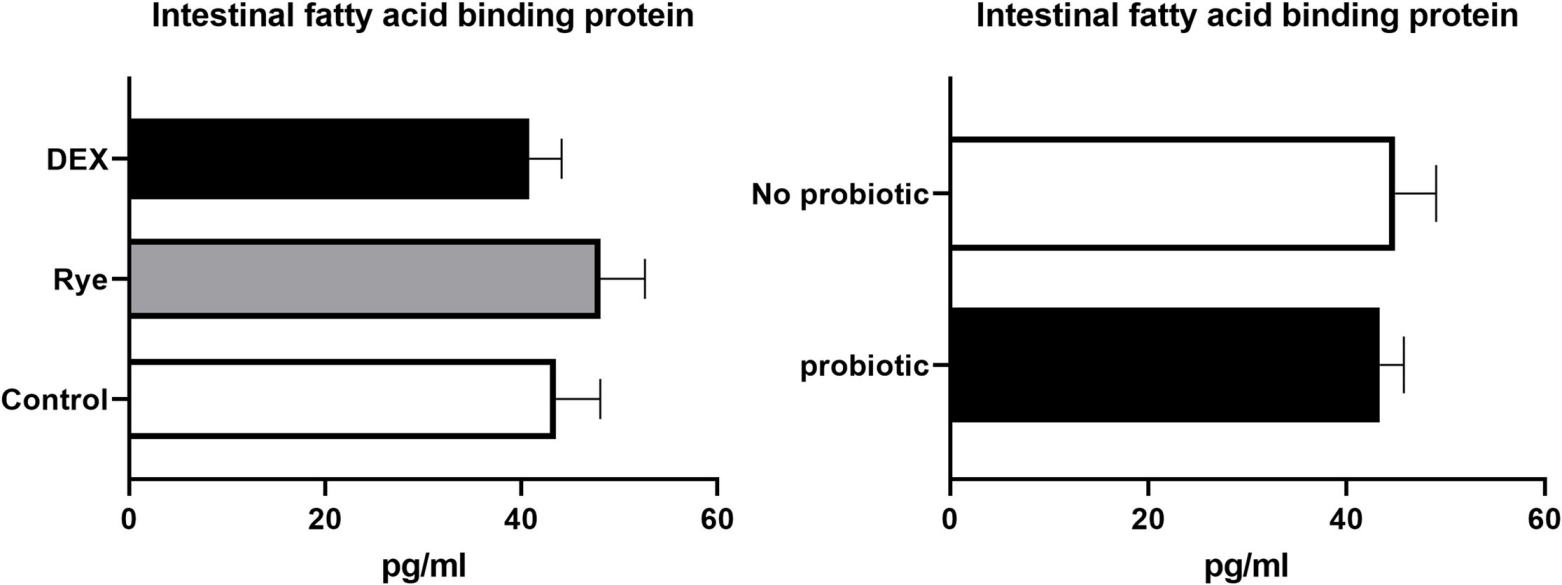
Concentration of intestinal fatty acid binding protein in excreta samples (n = 72) for the main effects of gut barrier dysfunction models (*P*>0.05) and probiotic supplementation (*P*>0.05). Error bars represent standard error of the mean (SEM).

**Fig 5:**
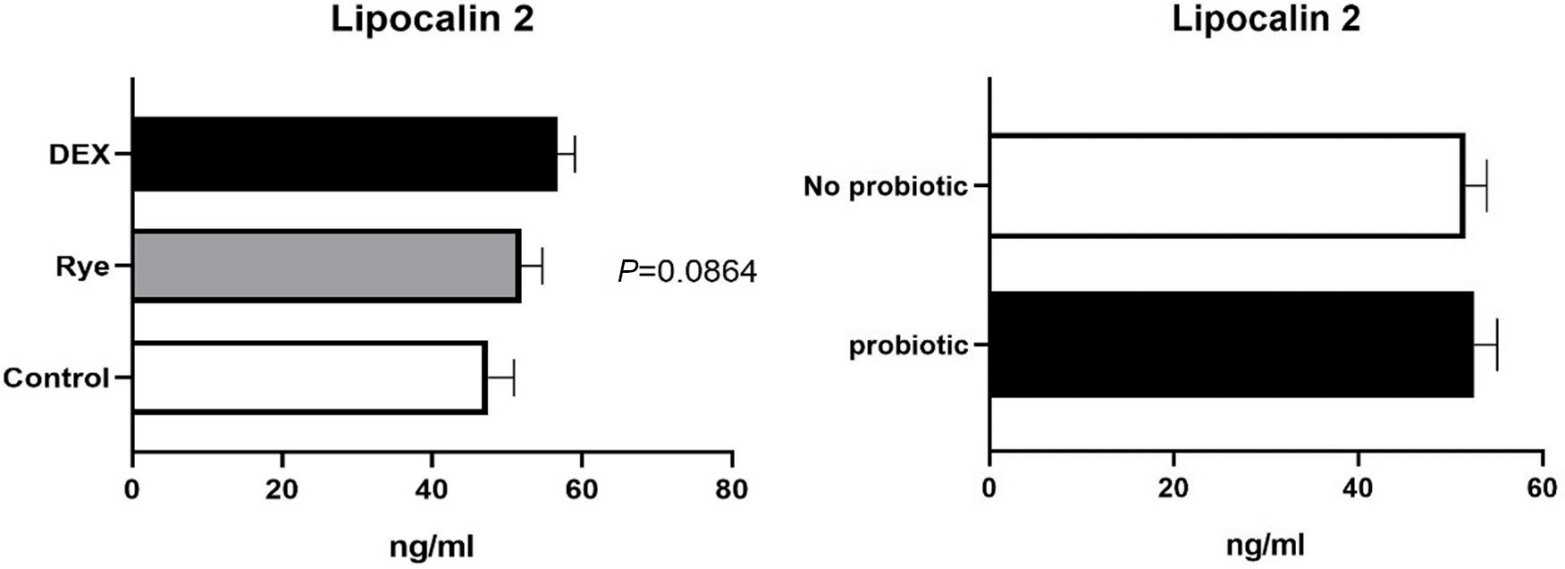
Concentration of Lipocalin 2 in excreta samples (n = 72) for the main effects of gut barrier dysfunction models and probiotic supplementation. Error bars represent standard error of the mean (SEM).

**Fig 6:**
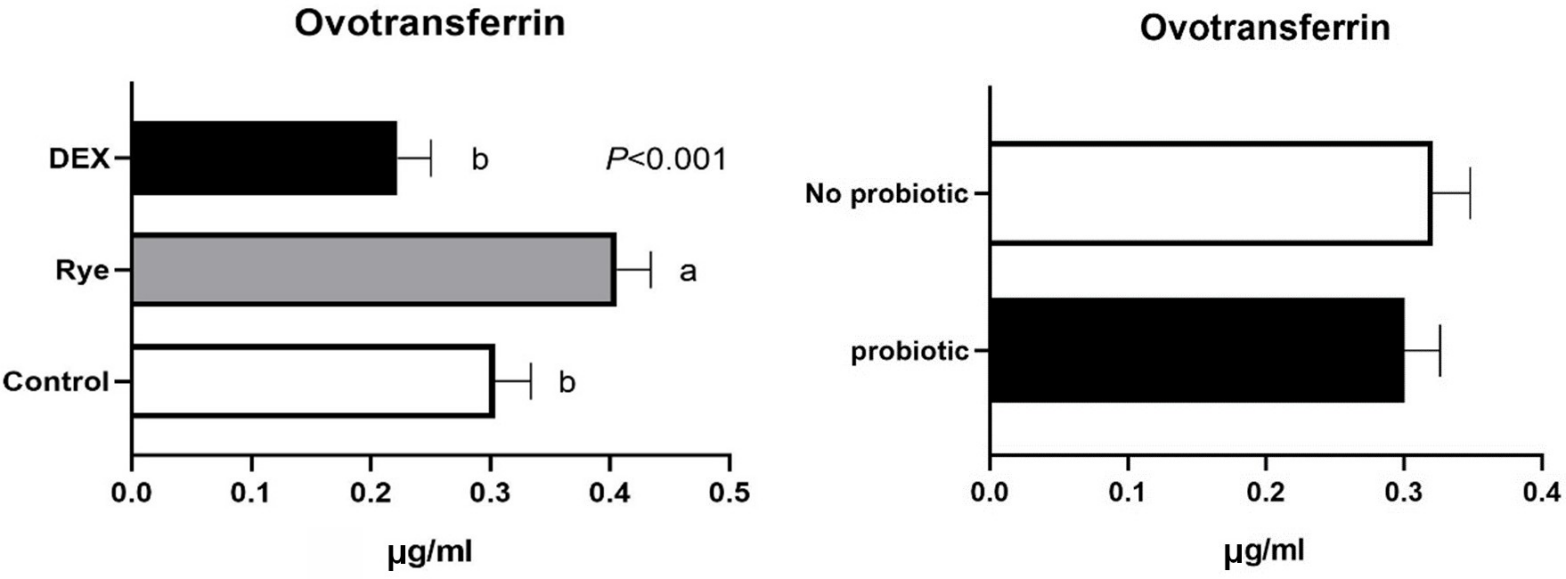
Concentration of ovotransferrin in excreta samples (n = 72) for the main effects of gut barrier dysfunction models and probiotic supplementation. Error bars represent standard error of the mean (SEM).

**Fig 7:**
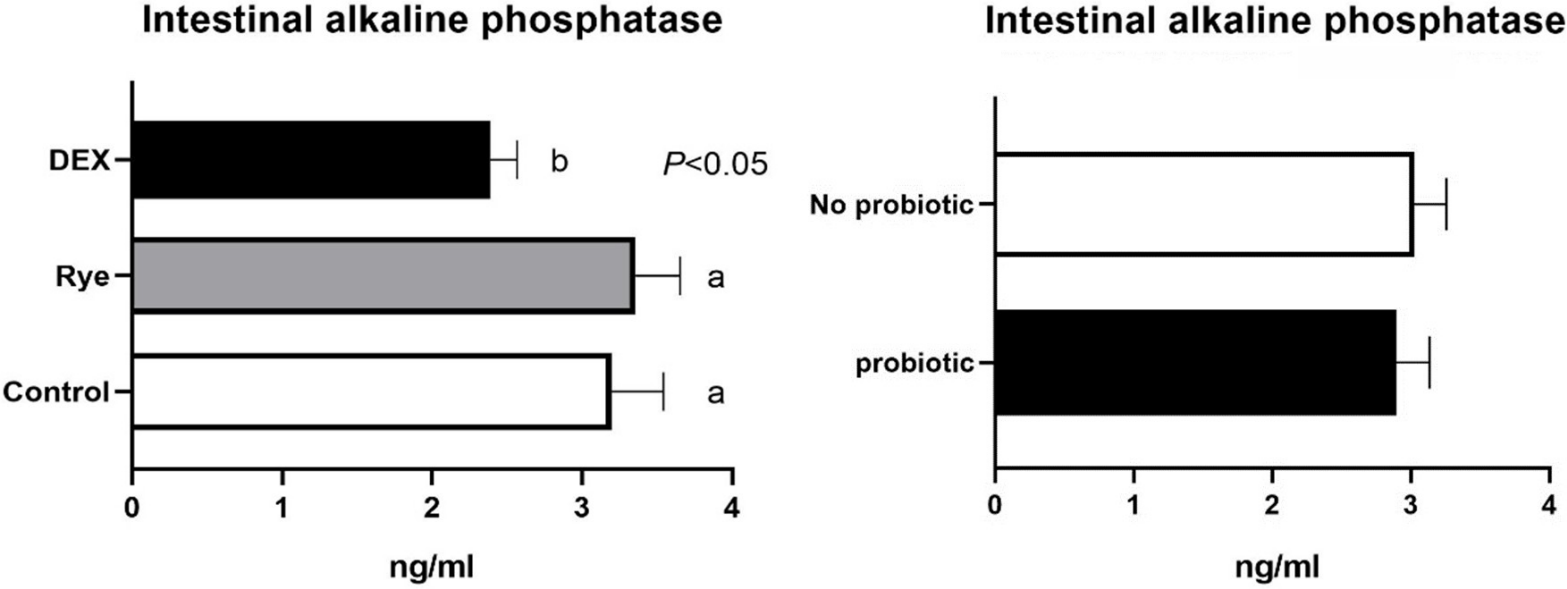
Concentration of intestinal alkaline phosphatase in excreta samples (n = 72) for the main effects of gut barrier dysfunction models and probiotic supplementation. Error bars represent standard error of the mean (SEM). Bars with differing superscripts are statistically different (*P*<0.05).

**Fig 8:**
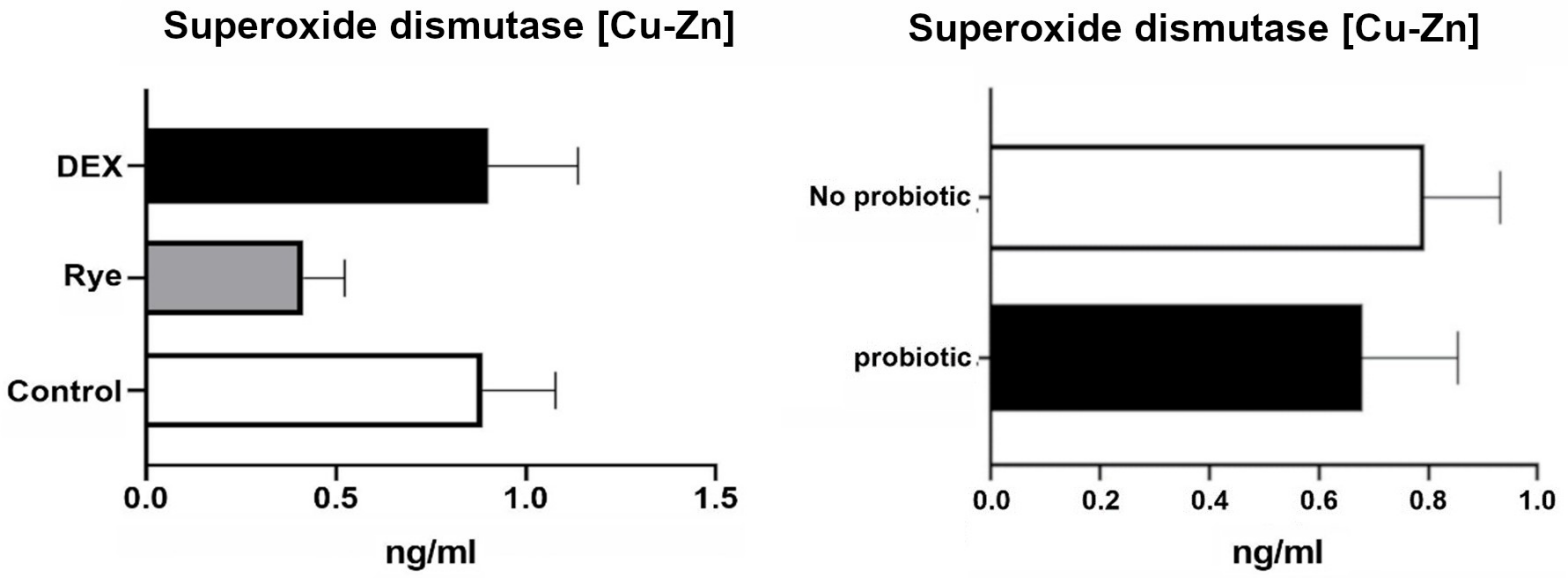
Concentration of superoxide dismutase [Cu-Zn] in excreta samples (n = 36) for the main effects of gut barrier dysfunction models (*P*>0.05) and probiotic supplementation (*P*>0.05). Error bars represent standard error of the mean (SEM).

The concentration of intestinal fatty acid binding protein in excreta remained similar for the experimental treatments showing an average of 44.1 pg/ml (Fig 4). As illustrated in Fig 5, lipocalin-2 tended (*P* = 0.086) to increase in excreta of birds injected with DEX compared with the control and rye diet. Compared to control birds, a marked 34% elevation (*P*<0.001; 0.406 vs 0.304 μg/ml) in excreta ovotransferrin was observed in birds fed the rye-based diet (Fig 6). There was no significant difference between DEX and control for ovotransferrin. Illustrated in Fig 7, injection of birds with DEX significantly decreased intestinal alkaline phosphatase (*P*<0.05) in excreta by 25% and 29% compared with control and rye-fed birds, respectively. As shown in Fig 8, the superoxide dismutase in excreta was not affected by the experimental factors showing a high variability (SD = 0.67) with an average concentration of 0.73 ng/ml.

## Discussion

Two different models were used to induce gut leakage in chickens in order to study a suite of potential biomarkers in excreta and a possible counteracting effect of a probiotic product with anti-inflammatory properties. Dexamethasone successfully induced gut leakage demonstrated by an increase in serum FITC-d consistent with recent observations [17, 20]. Such increased permeability, along with distinct retardation in growth and atrophy of immune organs, unequivocally supports that glucocorticoids (GC) have profound effects on gut barrier function. The mechanisms by which DEX can stimulate stress and impact gut integrity are mainly through GC type 1 receptors, mobilising glucose, and immunosuppression [21]. In the current study, the rye-based diet however failed to increase FITC-d concentration in serum contrary to other studies [22, 23]. Notably, in the current study a wheat-based control diet was used which may have diminished the effect of rye as a less digestible and rich source of non-starch polysaccharides (NSP). In hindsight, a maize-based control diet known to have less soluble NSP content may have provided a better opportunity to detect the permeability effects for this particular model. Nevertheless, both DEX [17, 24] and a rye-based diet [25] have been shown to increase intestinal permeability in broiler chickens compared with a wheat-based diet.

Both positive responses [26] and a lack of response [27] to probiotics for intestinal barrier function have been documented. The positive effect of probiotics is believed to be associated with changes in microbiota. The absence of a probiotic effect in the current study may be explained by the relatively short period of experimentation, housing conditions (i.e cage vs floor) and the basal diet composition and possibly viable organisms of the tested probiotic. Besides, the strain dependency and non-ubiquitous nature of the effects of probiotics on intestinal barrier function [13] may be other factors to explain the lack of effect in the present study. Given the absence of response to probiotics for all the parameters tested in the study, the discussion is mainly focused on the tested biomarkers in response to the gut barrier dysfunction models.

Fatty acid binding proteins (FABP) are molecules that coordinate lipid responses in cells and are known to be involved in metabolic and inflammatory pathways [28]. There are several FABP in different tissues including liver, intestine, heart, adipocyte and brain. Intestinal FABP (I-FABP) expressed in the intestine, is also known as FABP-2 and a potential candidate for intestinal barrier function. It has been shown in other species including mice that the expression of I-FABP occurs in every section of the intestine, but most abundantly in the distal region of the small intestine [28]. It appears that I-FABP can be thermostable with a denaturation point of 69°C in rat studies [29]. With mucosal damage and subsequent “leaky gut”, I-FABP can leak into the circulation from the epithelium leading to an increased concentration in plasma and subsequently voided in urine [30]. Very little research on the leaky gut and IFABP has been conducted in poultry, limited to gene expression in the intestine or plasma concentration [31]. In the current study, it was hypothesised that I-FABP could be detected in the excreta of poultry and used as a non-invasive tool to detect intestinal inflammation and permeability. Despite successful quantification of I-FABP in excreta samples, there was no significant difference between any of the experimental treatments in the current study. Possibly, I-FABP could be a faecal biomarker under more severe intestinal damage conditions [32] compared to the models tested in the current study. The relatively large molecular size of I-FABP, being around 15000 Da [33], may have prevented its passage through tight junctions or transcellular pathways at a high rate, as opposed to FITC-d with a much smaller molecular size (4000 Da). This may be a possible explanation as to why no changes in I-FABP were observed, even in birds injected with DEX; therefore, its potential as a biomarker in poultry warrants further research.

Alpha 1-antitrypsin is produced by the liver and is present in serum. This protein is relatively thermostable at room temperature [34], resistant to proteolysis in the intestine and reflects the loss of proteins to the intestinal lumen [35]. Alpha 1-antitrypsin concentration increases in the gut under conditions of increased permeability or when the mucosal barrier is disrupted. This is through extravasation from serum into the gut and ultimately in faecal material, making it a viable candidate as a biomarker of intestinal permeability [36]. We could not substantiate any differences between the treatments in relation to the concentration of alpha 1-antitrypsin in excreta of chickens. In accordance with this result, Gilani et al. [37] failed to find an association between concentration of alpha 1-antitrypsin with two gut leakage models caused by fasting and dextran sodium sulfate administration in chickens. It therefore appears that alpha 1-antitrypsin is not responsive to different models available in poultry and that the suitability of this protein for a non-invasive assessment of gut barrier dysfunction is questionable.

Lipocalin 2 (LCN2) is a glycoprotein proven to be a sensitive biomarker of various metabolic and inflammatory diseases as well as intestinal inflammation in rats and humans. LCN2 is a protein that limits bacterial growth by sequestering iron in the gut environment [9]. The concentration of LCN2 is typically low in biological fluids but elevated under inflammatory conditions [38]. The concentration of LCN2 is elevated in faecal material of mice subjected to dextran sulfate sodium induced colitis [39] or when fed high-fat and salt diets [40]. LCN2 is expressed in neutrophils, and in high permeability situations it can leak into the intestinal lumen from activated neutrophils making it a suitable faecal biomarker for non-invasively assessing inflammation and permeability [41]. LCN2 has not been studied in poultry as an excreta biomarker of barrier dysfunction although it is shown to be expressed in chickens [42]. The observed tendency for elevated LCN2 in excreta of birds under DEX injections could simply indicate the extensive effect of GC on intestinal barrier function as well as potential for this glycoprotein to be used as a biomarker of intestinal inflammation in poultry. DEX has been shown to upregulate the expression of LCN2 in murine chondrocytes [43]. Nevertheless, further verification is warranted in future experiments using different models such as heat stress or necrotic enteritis.

Ovotransferrin is an acute phase protein and its elevated levels can be used as a biomarker of inflammation in poultry in response to various inflammatory states induced by chemical, bacterial or viral factors [44]. It is believed that the loss of plasma proteins into the gastrointestinal tract is linked with disturbance of the intestinal barrier. In the current study the excreta concentration of ovotransferrin was elevated in birds fed a rye-based diet compared with other treatments. This result suggests a possible loss of intestinal integrity as a result of feeding high levels of rye and consequences on systemic inflammation. The elevated ovotransferrin in faecal material has recently been shown in response to necrotic enteritis [10] making it a worthwhile candidate for future validation studies. It is noted that activity of ovotransferrin is decreased by increasing the temperature [10].

Superoxide dismutase, an antioxidant enzyme, was assessed as a potential biomarker in excreta as this enzyme is responsive to oxidative stress and subsequent damage to intestinal barrier integrity. DEX is known to induce oxidative stress [45], and therefore it would be prudent to expect a change in Superoxide dismutase levels. However, the lack of differences in superoxide dismutase in the present study may be explained by considerably high variation in data obtained from individual birds for this particular assay, which can highlight a need for an increased in number of samples in any subsequent study. Assessed in serum samples of broilers, Baxter et al. [11] also found no change in superoxide dismutase in response to a rye-based diet using as a leaky gut model.

Fibronectin (FN) is a ubiquitous extracellular matrix (ECM) glycoprotein involved in tissue integrity through cell adhesion, proliferation and migration and is produced by multiple cell types [46] including intestinal epithelial cells [47]. It has been documented that FN levels are altered in patients suffering from ulcerative colitis or Crohn’s disease with major involvement in wound healing processes [47]. FN in its soluble form can be found in body fluids and in its insoluble form in the basement membrane and ECM of the intestinal wall [4]. Host mucosal damage can expose ECM, and protein such as FN can be released into intestinal contents and eventually excreta. In the case of inflammation or chronic injury, FN is expected to increase and therefore can be a potential biomarker for intestinal inflammation [47]. In the present study, the elevated concentration of FN in excreta of birds that received repeated DEX injections is in agreement with increased permeability and intestinal barrier failure of these birds, likely resulting from intestinal damage. Indeed, it has been shown that DEX can stimulate expression of FN [48]. Consistent with our results, recently De Meyer et al. [4] found a higher level of FN in colonic contents, as a proxy to excreta content, in birds challenged with a gut leakage model caused by necrotic enteritis.

The present study is the first report of elevated FN on actual excreta samples of broiler chickens in response to both nutritional and physiologically induced gut leakage models. Indeed FN is shown to be a stress responsive protein [46] and the stress stimulated by DEX in the present study further supports the idea that this protein may be a suitable biomarker of intestinal barrier failure and inflammation under stress conditions. The lower FN content of excreta in birds ingesting a rye-based diet compared with control birds cannot simply be explained by the data of the current study. However, it is probable that the nutrient composition, in particular carbohydrates, of wheat vs rye, or a possible negative effect on energy utilisation [49] may have contributed to the observed differences in FN. Despite the lack of any demonstrable effect of probiotic in the present study, ECM binding ability through its proteins, particularly FN, is worthy of future consideration for efficacy of selected probiotic strains, in particular, *Lactobacillus sp*. [50]. Probiotic bacterial strains can compete with pathogenic bacteria for binding receptors such as FN [51] and therefore under an unfavourable increase of FN, the presence or supplementation of a probiotic may be beneficial. Nevertheless, further studies are required to establish a range of FN concentrations in poultry; with any abnormal concentration presenting an opportunity for diagnosis and progression of particular intestinal disorders.

Enterocytes secrete intestinal alkaline phosphatase (IAP) both apically and basolaterally. IAP plays a pivotal role in regulation of bicarbonate secretion, absorption of long chain fatty acids and mitigation of intestinal inflammation, as well as influencing both composition and translocation of the gut microbiota [52]. Thomas and Henton [12] were the first to propose IAP as a faecal biomarker of intestinal damage in rats. In poultry, data for IAP as a faecal biomarker are indeed scarce. Nevertheless, when intestinal damage occurs, digestive enzyme secretion may reduce, thereby making IAP a viable biomarker [5]. In the present study, DEX significantly reduced IAP in the excreta compared to control and rye fed birds. Although there is no directly comparable study in the literature, the activity of IAP has been shown to decrease in broilers under heat stress [53]. There may be a few possible explanations for alteration in IAP. Firstly, possible damage to enterocytes caused by DEX-stress stimulated may have negatively affected secretion of IAP. Secondly, repeated GC exposure may increase intestinal permeability which eventually can make the birds susceptible to mucosal inflammation [20]. Thirdly, the lower IAP may also, at least in part, explain the impaired intestinal barrier function in DEX injected birds with recent evidence of IAP impacting key tight junction proteins [54]. However, it should be noted that depending on the type of AP isoforms, both upregulation or down regulation of IAP is possible in response to inflammation or a metabolic disorder. The inflammation may lower the IAP concentration but not tissue non-specific AP isoforms [52]. Therefore, it is likely that IAP measured in the current study originated from the small intestine. A caveat in using IAP as a biomarker may be the confounding factors such as differences in feed intake or dietary composition that can change intestinal production and release of IAP [55]. The potential of IAP as a biomarker and a potent controlling agent for intestinal inflammation and barrier function warrants further research in poultry.

Individual values within treatments showed considerable variation for several biomarkers, which could have been reflective of several factors including intra- and inter-ELISA assay variations, natural biological variation between individual animals, or the sample homogeneity. In future studies it would be worthwhile to compare excreta samples from group-housed birds with those obtained from individual animals or at a flock level. Ideally, for diagnostic purposes, biomarkers should demonstrate the least overlap between birds with compromised gut barrier and healthy birds. Regarding the stability of tested biomarkers, the samples used in the current study were collected from birds 6 hours after trays were cleaned to allow time for defecation and collection of sufficient and representative samples in a non-invasive method. Although protein stability may have been affected during the collection procedure, for all the biomarkers, samples were treated identically. As a result, any differences associated with the collection time would likely have been reflected for all the samples, and not necessarily for a particular treatment. Nevertheless, thermal stability of the biomarkers should be investigated in future studies.

## Conclusions

Detection of a gut health problem at an early stage benefits the poultry industry through reducing cost of poor enteric health and associated compromised performance. This will allow rapid intervention to address the issue through management strategies, feed additives or seeking veterinary advice. Subject to further validation studies, the poultry industry can adapt the results of this study by adapting rapid testing through sample screening at farm level or developing devices that can detect the intensity of a particular biomarker on a real-time basis. It appears that ELISA with careful set up and modification can be a useful test particularly when reliable commercial kits are made available.

In quest of suitable biomarkers of intestinal barrier function across species, there is a consensus that a suite of multiple biomarkers is superior to any single one to represent the status of a gut integrity related issue [5, 56]. Similarly in the current study, it appeared that the response of biomarkers to different gut leakage models may differ, which further emphasises the use of multiple biomarkers. The present study identified candidate biomarkers that could be detected in the excreta of broiler chickens as a non-invasive method to assess gut barrier function and inflammation. Among tested biomarkers, FN and IAP were found to be responsive to stress induced by DEX and were consistent with results obtained for permeability as assessed by FITC-d concentration in blood samples. In addition, for the first time a similar trend was also identified for LCN2 in this study. Future validation studies at flock level with consideration to litter samples and sample homogeneity are required in order to facilitate the use of biomarkers in the poultry industry.

## Supporting information

S1 File Raw data and scatter graphs for individual data. (XLSX)
